# Tumor Vascularity and Glucose Metabolism Correlated in Adenocarcinoma, but Not in Squamous Cell Carcinoma of the Lung

**DOI:** 10.1371/journal.pone.0091649

**Published:** 2014-03-10

**Authors:** Jiuquan Zhang, Lihua Chen, Yongfeng Chen, Wenwei Wang, Lin Cheng, Xiangdong Zhou, Jian Wang

**Affiliations:** 1 Department of Radiology, Southwest Hospital, Third Military Medical University, Chongqing, China; 2 Department of Respiratory Medicine, Southwest Hospital, Third Military Medical University, Chongqing, China; Northwestern University Feinberg School of Medicine, United States of America

## Abstract

**Background/Objectives:**

To prospectively examine the relation between tumor vascularity and glucose metabolism in adenocarcinoma (AC) and squamous cell carcinoma(SCC) of the lung by using positron emission tomography/computed tomography (PET/CT) and dynamic contrast enhanced magnetic resonance imaging (DCE-MRI).

**Materials and Methods:**

Forty-one consecutive patients with histologically confirmed untreated NSCLC underwent routine diagnostic work-up, including DCE-MRI and PET/CT. PET/CT images were used to derive glucose metabolism (SUVmax and SUVmean), and DCE-MRI images were used to derive tumor vascularity (Ktrans, Kep, Ve and iAUC). Any differences in the DCE-MRI and PET/CT estimations between the NSCLC subtypes were determined by the Wilcoxon rank sum test. Spearman’s rank correlation coefficients were calculated between the DCE-MRI parameter values and the SUV.

**Results:**

SUVmean and SUVmax in AC were significantly lower than in SCC, but Ktrans and Ve in AC were significantly higher than in SCC. Significant correlations between SUV and DCE-MRI parameters were observed for SUVmax and Ve (ρ = −0.357, P = 0.022), SUVmean and Ktrans (ρ = −0.341, P = 0.029), and SUVmean and iAUC (ρ = −0.374, P = 0.016 ) in total; for SUVmax and iAUC (ρ = −0.420, P = 0.037), SUVmean and Ktrans (ρ = −0.411, P = 0.041), SUVmean and Kep (ρ = −0.045, P = 0.026), and SUVmean and iAUC (ρ = −0.512, P = 0.009) in AC; However, for neither in SCC.

**Conclusion:**

AC and SCC showed different patterns in both tumor vascularity and glucose metabolism. Tumor vascularity and glucose metabolism negatively correlated in AC, but not in SCC. These differences may underlie the heterogeneity in clinical aspect of NSCLC subtypes and have implications for their imaging profiling and monitor the treatment response.

## Introduction

For therapeutic purposes, non-small cell lung cancer (NSCLC) has traditionally been regarded as a single disease. However, recent evidences suggest that the two major subtypes of NSCLC, adenocarcinoma (AC) and squamous cell carcinoma (SCC), are heterogeneous in many clinical aspects. AC responds to chemotherapy better than SCC [1], but it has a greater tendency to relapse in the form of distant metastases than SCC [1]. After surgical resection, AC has higher rates in recurrence and mortality than SCC [2] in Western countries, but in East Asia AC has better prognosis [3]. Fundamental discrepancies in tumor biology of NSCLC subtypes may be a primary factor determining the differential clinical manifestation.

Both tumor vascularity and glucose metabolism are important aspects of the tumor biology. Angiogenesis, the sprouting of new capillaries from existing blood vessels, and vasculogenesis, the de novo generation of blood vessels are the two primary methods of vascular expansion by which nutrient supply to tissues is adjusted to match physiological needs. Pathological angiogenesis is critical for growth and metastasis of malignant tumors [4]. The phenomena known as the ‘Warburg Effect’ was described by Otto Warburg during his lifetime of work into cellular metabolism and respiration [5]. He recognized that glucose can be metabolized either by combination with oxygen, i.e. respiration, or by glycolysis to produce lactate. He also observed that a change from oxidative phosphorylation to the less energy efficient glycolysis, even in the presence of an adequate supply of oxygen, is a fundamental property of the metabolism of cancer cells and that the rate of glycolysis correlated with tumor growth. Today, Warburg’s findings underpin the principles of tumor imaging with fluorodeoxyglucose positron emission tomography (FDG-PET) [6].

Although tumor vascularity and glucose metabolism tightly coupled in most normal tissues, many studies have shown that the relationship between vascular physiology and glucose metabolism is not well matched in tumors [7]. The balance between tumor blood flow and metabolism will be an important indicator of the biological status of a tumor and hence the tumor’s likely progression and response to treatment [6].

There is an increasing opportunity to perform multifunctional imaging at a variety of organ sites with relatively short examination times. Each technique yields quantitative parameters that reflect specific aspects of the underlying tumor or tissue biology [7]. Dynamic contrast enhanced magnetic resonance imaging (DCE-MRI) using small molecular weight gadolinium chelates enables non-invasive imaging characterization of tissue vascularity. FDG-PET creates tomographic images that represent glucose metabolic activity of underlying tissue processes.

Thus, our aim was to compare the tumor vascularity and glucose metabolism parameters and to explore the relationship between them in patients with different subtypes of NSCLC by using DCE-MRI and PET/CT.

## Materials and Methods

### Patients

Forty-one consecutive patients with histologically confirmed untreated NSCLC (AC, n = 25; SCC, n = 16)underwent routine diagnostic work-up, including DCE-MRI, and a combined PET/CT whole body imaging (median interval between the two examinations 1 day, range 0–5 days). The clinical characteristics are summarized in Table 1. The UICC International Union Against Cancer TNM system (7th edition) was used for staging [8].

**Table 1:** Clinical characteristics of included NSCLC patients.

	No. of patients	sex	age	longest diameter(cm)	volume(ml)	clinical stage
All	41	16F, 25M	56.463±12.808	5.754±1.291	101.060±60.808	I b (n = 4) II(n = 4 )
	–	–	(29, 80)	(3.000, 7.900)	(52.970, 272.970)	III (n = 12) IV(n = 21 )
AC	25	15F, 10M	57.800±13.441	5.780±1.076	100.018±45.088	I b(n = 2) II (n = 1)
	–	–	(29, 76)	(3.900, 7.900)	(52.970, 225.850)	III (n = 8) IV(n = 14 )
SCC	16	1F, 15M	54.375±11.865	5.713±1.609	102.688±81.259	I b(n = 2) II(n = 3 )
	–	–	(33, 80)	(3.000, 7.500)	(14.870, 272.970)	III (n = 4 ) IV(n = 7 )
P value	–	–	0.316	0.708	0.471	0.470

Data are reported as mean ± SD with minimum and maximum in parentheses.

The Medical Research Ethics Committee of the Third Military Medical University (Chongqing, China) reviewed and approved the present study. Written informed consent was obtained from each participant prior to the study.

### MRI Examinations

In all patients, MRI was performed on a 3.0 T scanner (Magnetom Trio; Siemens Medical Solutions, Erlangen, Germany). Axial turbo inversion recovery magnitude (TIRM) imaging, T2-weighted imaging (TR (repetition time) 3800 ms, TE (echo time) 80 ms, FOV 380×285 mm, matrix 320×164), and volumetric interpolated breath-hold examination(VIBE), T1-weighted imaging (TR 3.78 ms, TE 1.66 ms, FOV 400×262 mm, matrix 384×190), as well as coronal T1- weighted imaging were acquired before contrast agent administration. For conversion of the signal intensities into gadolinium concentration a T1 map was used. The T1 map was calculated from pre-contrast dual flip angle images (2° and 15°) [9]. After gadolinium (Magnevist, Bayer Schering Pharma, Berlin, Germany) injection (0.2 mmol/kg; mean dose of the administered gadolinium 12±1.7 ml, range 10–17 ml, flow rate 3 ml/s), using a power injector, dynamic coronal VIBE T1-weighted images (TR 3.63 ms, TE 1.29 ms, number of averages 1, FOV 420×341 mm, matrix 224×182, flip angle 12°, 20 slices, slice thickness 4 mm, generalized auto-calibrating partially parallel acquisition (GRAPPA) accelerator factor 2, measurements 100, total acquisition time 5 min 59 s.) were acquired. To minimize the influence on data quality of respiratory movement, several approaches have been taken: (1) Breath-hold for the pre-contrast dual flip angle images and first pass (∼20 s) then allow breathing; (2) Guided free breathing (instructions from the imaging radiographer).

### Whole Body 18F-FDG PET/CT Examinations

After a fasting period of at least 6 h and with the precondition of a blood glucose level below 130 mg/dl, the patients were intravenously injected with 3 MBq [18F] FDG per kilogram body weight and 20 mg furosemide for reduction of radiation exposure to the bladder wall. They were asked to stay in a semi-Fowler’s position in a quiet and darkened room for 60 min. [18F] FDG imaging was performed on an integrated PET/CT system (Biograph 6 TruePoint PET/CT, Siemens Medical Solutions, Erlangen, Germany). Contrast-enhanced CT images were initially obtained from the top of the skull to the proximal thighs (120 mAs, 130 kV, 4-mm slice collimation) followed by PET in 3D mode with an acquisition time of 3 min per bed position (axial FOV 16.2 cm). Images were reconstructed by an attenuation-weighted ordered-subsets expectation maximisation algorithm (four iterations, eight subsets) and a post-reconstruction smoothing Gaussian filter. The CT images in the chest were reconstructed in 4-mm-thick slices in order to provide an easier comparison with the DCE-MRI slices.

### Data Analysis

Two senior radiologists, blinded to the PET/CT and histopathological findings, consensually reviewed tumor findings on MR images. The DCE-MR images were transferred for post-processing to a workstation running commercially available software for tissue perfusion estimation (Tissue 4D, Siemens Medical Systems). After motion correction and registration of the pre- and post-contrast acquisitions, T1 mapping was automatically performed and a freehand region-of-interest (ROI) was plotted around the tumor. Given the possible breathing artifacts, the outlier time point was discarded according to the fitting curve. The pharmacokinetic modeling was based on a two-compartment model that allows for the calculation of Ktrans, Ve, Kep (transfer constant from the extracellular extravascular space to plasma, where Kep = Ktrans×Ve^ −1^) and iAUC (initial area under the signal intensity–time curve) [10], [11]. Ktrans is a parameter related to vessel permeability and tissue blood flow, the leakage space Ve is a marker of cell density, and iAUC is related to the blood volume in the tissue of interest. For the subsequent tumor ROI analysis post-contrast T1-weighted as well as T2-weighted images were utilized to assist in the tumor delineation in all involved slices in order to avoid necrotic areas and large feeding vessels in close proximity. Additionally, according to previous reports [12], the ground glass appearances (a feature of adenocarcinoma) [13], [14] and necrosis (a feature of squamous cell tumors) [15] have potential impact on the DCE data measurement. To reduce this effect, in outlining the ROI for measurement the DCE metrics, we tried our best to draw in on the solid part of the tumors by integrating the multimodality imaging information including the conventional contrast-enhanced CT and/or MRI, PET/CT. The DCE-MRI parameters of each slice were recorded and were averaged in order to gain whole tumor DCE-MRI estimates.

Two observers, one senior radiologist experienced in chest imaging and a nuclear medicine specialist reviewed tumor findings on FDG-PET/CT images in consensus. Tumor tissue was identified as any voxel in the 3D dataset with counts greater than a fixed threshold fraction of the peak activity in the tumor [16]. The threshold level for tumor characterization was selected as mean SUV (SUVmean) equal to or higher than 2.5 [16]. The SUVs were calculated automatically by the software (TrueD, Siemens Medical Systems) using the body weight method: SUV = [decay corrected tissue activity (kBq ml^−1^)]/[injected ^18^F FDG dose per body weight (kBq g^−1^)]. The maximum SUV (SUVmax) and SUVmean of the tumor tissue were derived automatically by the software using a volume-of-interest (VOI) method of tissue delineation.

### Statistical Analysis

All statistical analyses were performed by using SPSS statistical software (Version 17.0; SPSS, Chicago, Illinois). Intraclass correlation coefficient (ICC) was used to determine the levels of interobserver variability in quantitative analysis of DEC-MRI and PET metrics. The values were presented as mean or median ± standard deviation (SD). The difference of clinical stage between the NSCLC subtypes was determined by Fisher exact test analysis. Any differences in the DCE-MRI and PET/CT estimations between the NSCLC subtypes were determined by the Wilcoxon rank sum test. Spearman’s rank correlation coefficients were calculated between the DCE-MRI parameter values and the SUV. The Bonferroni method was applied for multiple comparison correction [17]. A Bonferroni method corrected P value less than 0.05 was considered as significant.

## Results

### ICC Analysis

The ICCs between the two radiologists for the measurement of SUVmean, SUVmax, Ktrans, Kep, Ve and iAUC were 0.89, 0.93, 0.85, 0.82, 0.80, and 0.81, respectively. The final values were the means of the two measurements.

### Comparison of PET/CT and DCE-MRI Metrics between AC and SCC

**Figure 1:**
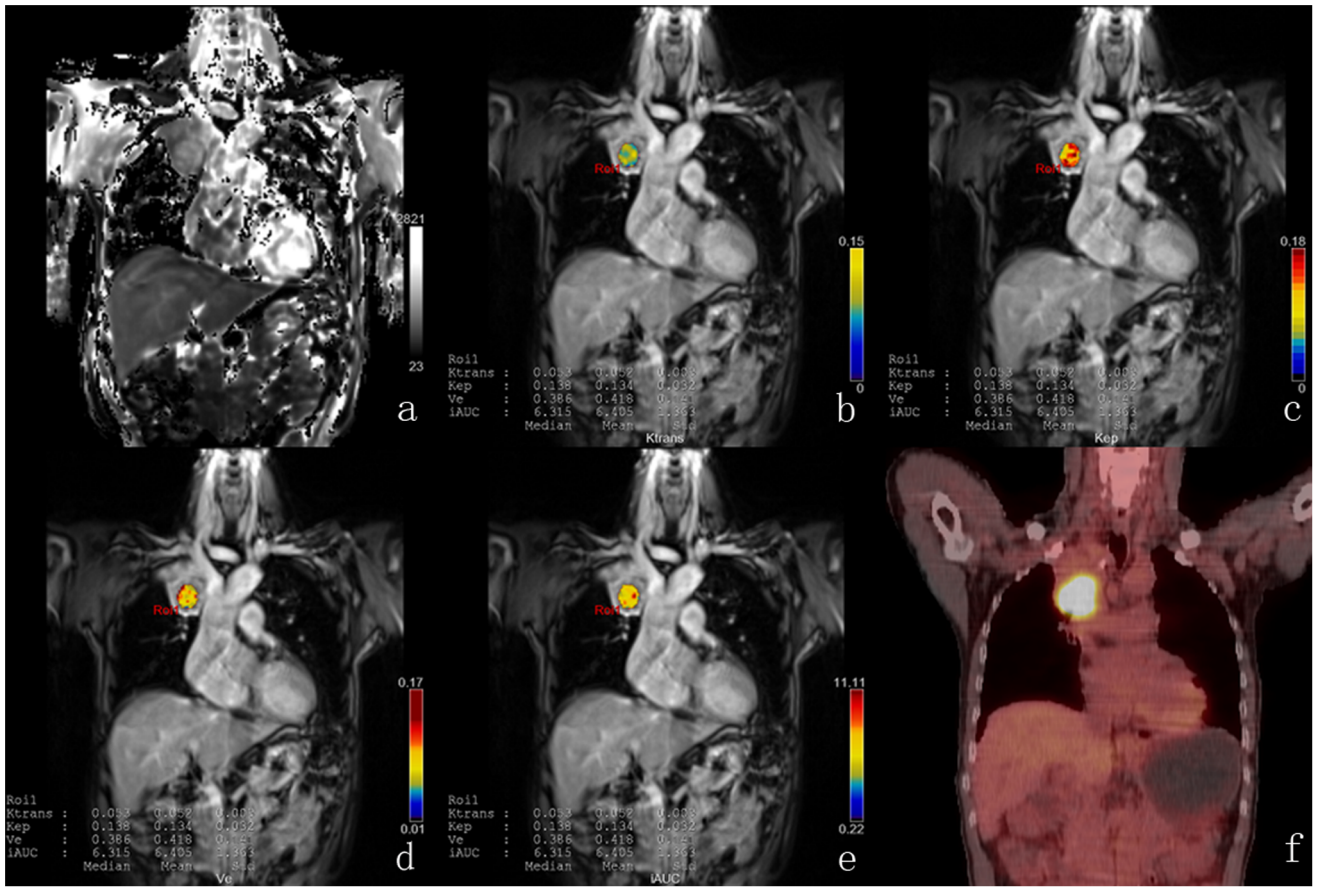
DCE-MRI and PET/CT of a representative case with SCC. T1 map (a) is automatically calculated by the precontrast images. Color parametric maps of Ktrans (b), Kep (c), Ve (d) and iAUC (e) based on coronal T1- weighted images of the chest demonstrate the increased tumor microcirculation parameters on the right upper lung. The corresponding PET/CT image (f) shows the avid glucose uptake in the tumor site.

**Figure 2:**
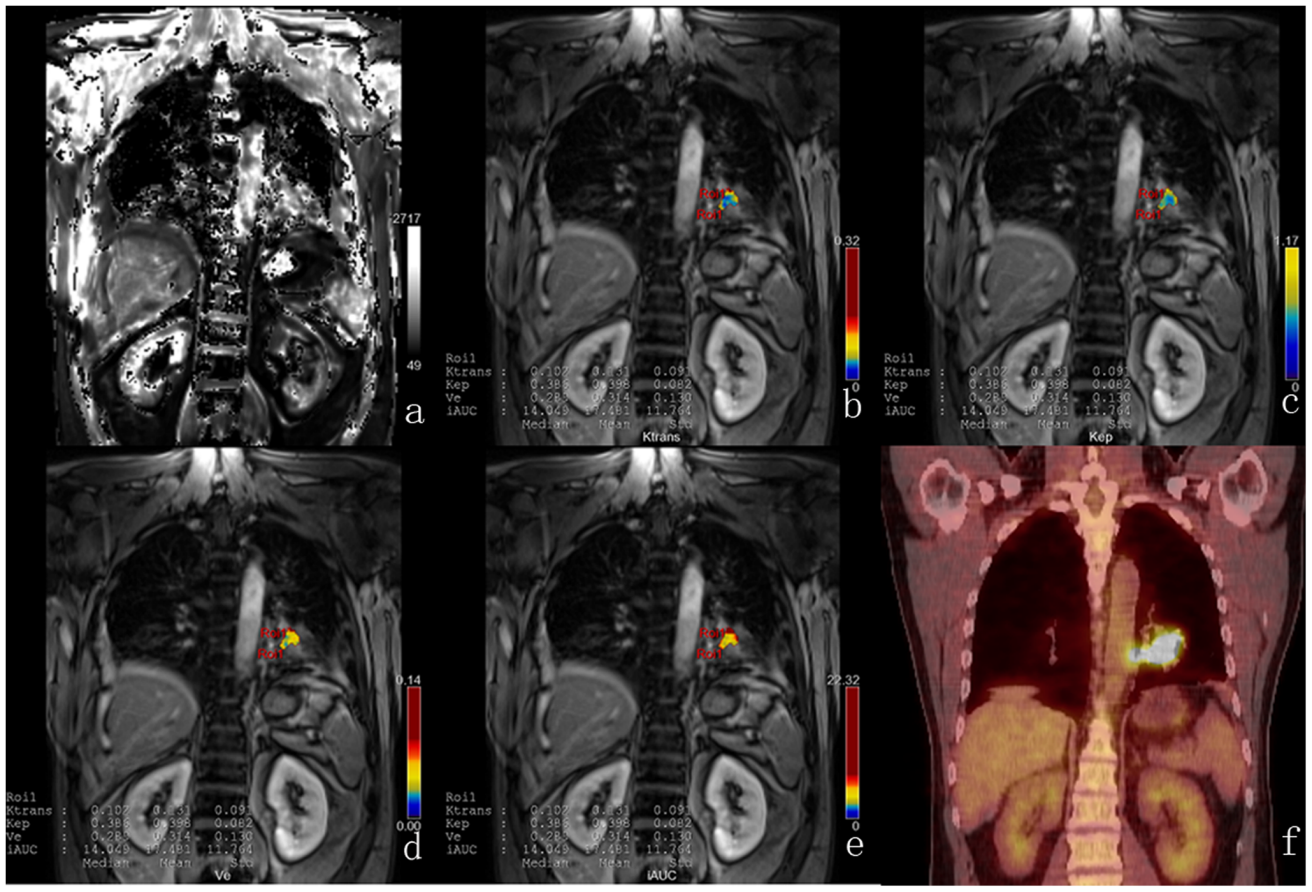
DCE-MRI and PET/CT of a representative case with AC. T1 map (a) is automatically calculated by the precontrast images. Color parametric maps of Ktrans (b), Kep (c), Ve (d) and iAUC (e) based on coronal T1- weighted images of the chest demonstrate the increased tumor microcirculation parameters on the left lower lung. The corresponding PET/CT image (f) shows the avid glucose uptake in the tumor site.

All PET/CT and DCE-MRI studies were acquired without side effects and were suitable for further evaluation. The summary statistics for the DCE-MRI and PET/CT metrics in the NSCLC subtypes are demonstrated in Table 2. SUVmean and SUVmax in AC were significantly lower than in SCC, but Ktrans and Ve in AC were significantly higher than in SCC (Table 2). A representative case with SCC of DCE-MRI and PET/CT imaging is shown in Fig. 1, with AC in Fig. 2.

**Table 2:** Summary statistics of the average SUV_max_ and DCE-MRI parameters in primary tumors of NSCLC and its subtypes.

	No. of patients	SUVmean	SUVmax	Ktrans (min^−1^)	Kep(min^−1^)	V_e_	iAUC (mM s)
All	41(16F, 25M)	5.300±1.343	12.077±4.795	0.093±0.057	0.359±0.117	0.279±0.149	13.107±7.846
		(4.876, 5.723)	(10.564, 13.591)	(0.075, 0.111)	(0.322, 0.396)	(0.232, 0.326)	(10.631, 15.583)
AC	25(15F, 10M)	4.824±0.970	10.376±3.069	0.104±0.058	0.351±0.124	0.313±0.147	14.442±8.000
	–	(4.424, 5.225)	(9.110, 11.643)	(0.080, 0.128)	(0.300, 0.403)	(0.252, 0.373)	(11.140, 17.744)
SCC	16(1F, 15M)	6.042±1.530	14.735±5.821	0.076±0.052	0.371±0.109	0.227±0.140	11.022±7.360
	–	(5.227, 6.857)	(11.633, 17.837)	(0.049, 0.104)	(0.313, 0.429)	(0.153, 0.302)	(7.100, 14.943)
P value	–	0.008*	0.015*	0.035*	0.250	0.042*	0.069

Data are reported as median ± SD with 95% CI in parentheses. The statistically significant differences in the functional estimates between AC and SCC are also indicated with asterisk.

### Correlation Analysis between PET/CT and DCE-MRI Metrics in AC and SCC

**Figure 3:**
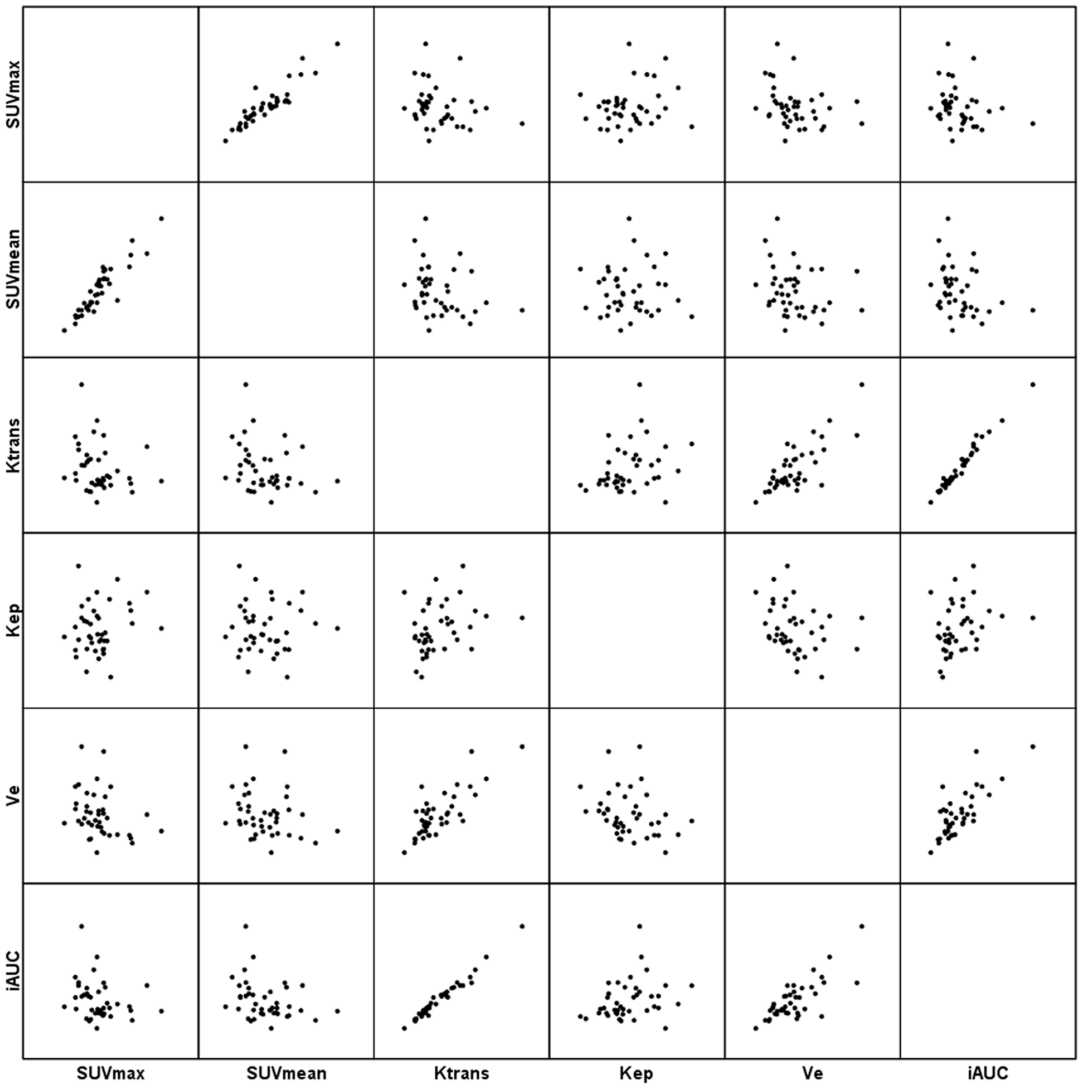
Scatter matrix of the PET/CT and DCE-MRI metrics in total NSCLC.

**Figure 4:**
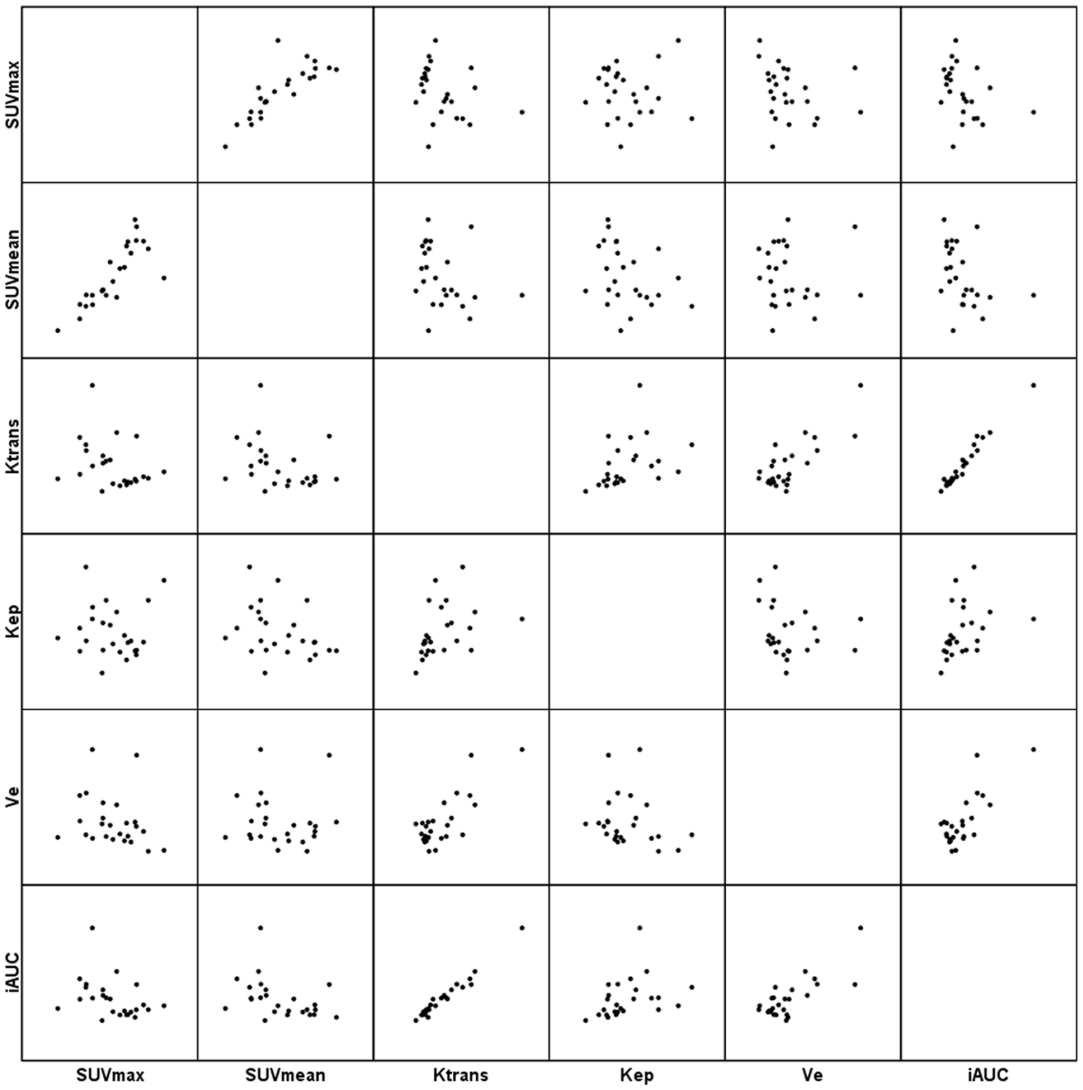
Scatter matrix of the PET/CT and DCE-MRI metrics in AC of the lung.

**Figure 5:**
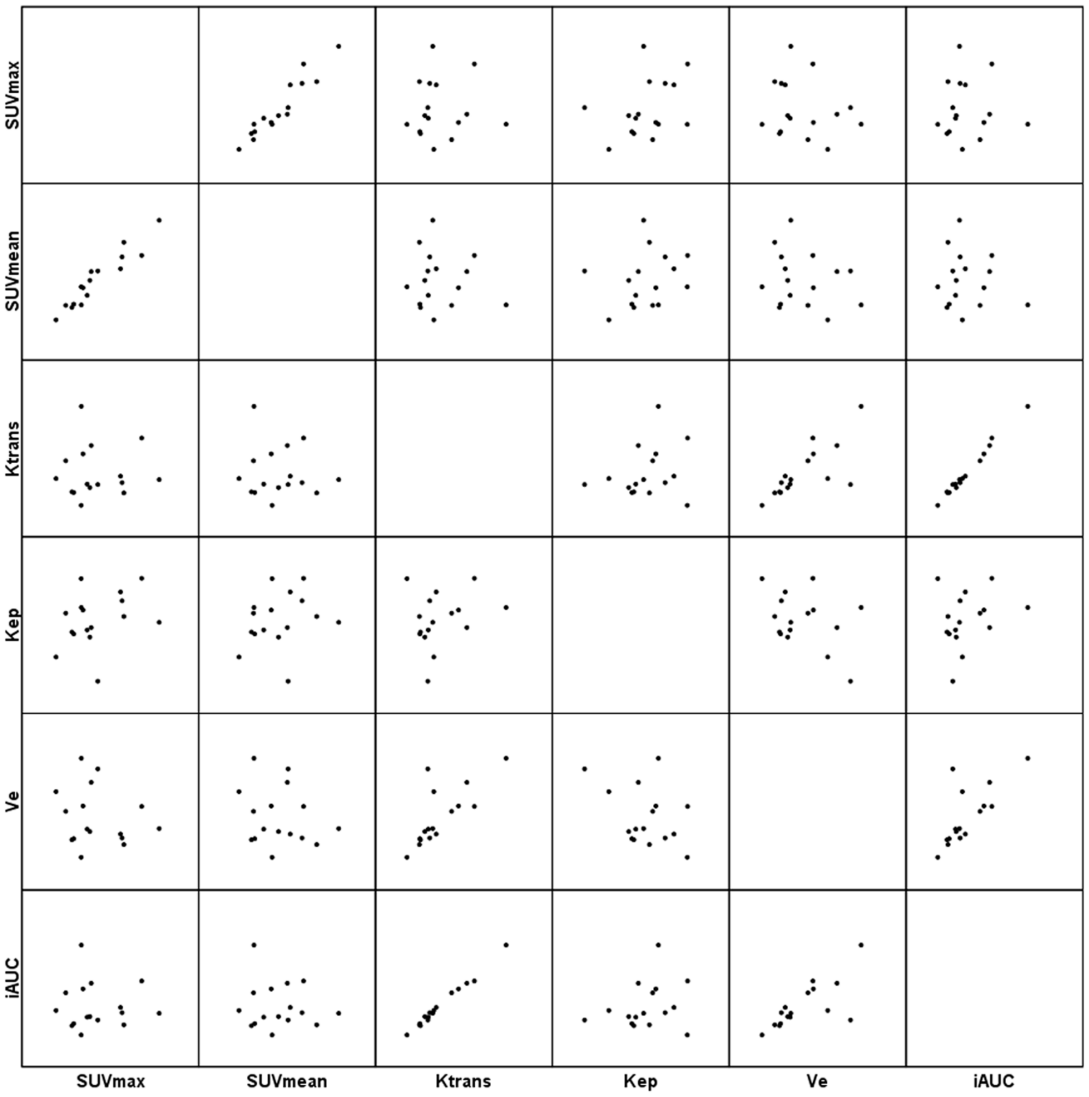
Scatter matrix of the PET/CT and DCE-MRI metrics in SCC of the lung.

The Spearman’s ρ coefficient analysis between the SUVmax and SUVmean in the tumor sites demonstrated significant correlations in total(Fig. 3), AC and SCC at a high level of statistical significance (ρ = 0.926, P < 0.001, Fig. 4; ρ = 0.867, P < 0.001 and ρ = 0.968, P < 0.001, Fig. 5, respectively). (Table 3).

**Table 3:** Correlation analysis of the DCE-MRI and SUV parameters in primary tumors of NSCLC and its subtypes.

		total		AC		SCC	
Variable 1	Variable 2	Spearman ρ	P value	Spearman ρ	P value	Spearman ρ	P value
Between SUV parameters							
SUVmax	SUVmean	0.926	0.000*	0.867	0.000*	0.968	0.000*
Among DCE-MRI parameters							
Ktrans	Kep	0.392	0.011*	0.606	0.001*	0.338	0.200
Ktrans	Ve	0.656	0.000*	0.537	0.006*	0.776	0.000*
Ktrans	iAUC	0.974	0.000*	0.962	0.000*	0.979	0.000*
Kep	Ve	−0.334	0.033*	−0.287	0.164	−0.185	0.492
Kep	iAUC	0.384	0.013*	0.566	0.003*	0.359	0.172
Ve	iAUC	0.637	0.000*	0.530	0.006*	0.738	0.001*
SUV vs. DCE-MRI parameters							
SUVmax	Ktrans	−0.277	0.080	−0.329	0.108	0.100	0.713
SUVmax	Kep	0.103	0.524	−0.129	0.539	0.318	0.231
SUVmax	Ve	−0.357	0.022*	−0.354	0.083	−0.021	0.940
SUVmax	iAUC	−0.300	0.056	−0.420	0.037*	0.124	0.649
SUVmean	Ktrans	−0.341	0.029*	−0.411	0.041*	−0.032	0.905
SUVmean	Kep	−0.050	0.757	−0.445	0.026*	0.350	0.184
SUVmean	Ve	−0.290	0.065	−0.107	0.612	−0.147	0.587
SUVmean	iAUC	−0.374	0.016*	−0.512	0.009*	0.009	0.974

The statistically significant correlations are indicated with an asterisk.

Significant correlations between DCE-MRI parameters were observed for Ktrans and Kep (ρ = 0.392, P = 0.011), Ktrans and Ve (ρ = 0.656, P < 0.001), Ktrans and iAUC (ρ = 0.974, P < 0.001), Kep and Ve (ρ = −0.334, P = 0.033), Kep and iAUC (ρ = 0.384, P = 0.013) and Ve and iAUC (ρ = 0.637, P < 0.001) in total(Fig. 3); for Ktrans and Kep (ρ = 0.606, P = 0.001), Ktrans and Ve (ρ = 0.537, P = 0.006), Ktrans and iAUC (ρ = 0.962, P < 0.001), Kep and iAUC (ρ = 0.566, P = 0.003) and Ve and iAUC (ρ = 0.530, P = 0.006) in AC(Fig. 4); and for Ktrans and Ve (ρ = 0.776, P < 0.001), Ktrans and iAUC (ρ = 0.979, P < 0.001), and Ve and iAUC (ρ = 0.738, P = 0.001) in SCC(Fig. 5). (Table 3).

Significant correlations between SUV and DCE-MRI parameters were observed for SUVmax and Ve (ρ = −0.357, P = 0.022), SUVmean and Ktrans (ρ = −0.341, P = 0.029), and SUVmean and iAUC (ρ = −0.374, P = 0.016 ) in total(Fig. 3); for SUVmax and iAUC (ρ = −0.420, P = 0.037), SUVmean and Ktrans (ρ = −0.411, P = 0.041), SUVmean and Kep (ρ = −0.045, P = 0.026), and SUVmean and iAUC (ρ = −0.512, P = 0.009) in AC(Fig. 4); However, for neither in SCC (Fig. 5). (Table 3).

## Discussion

Our results showed that both SUVmax and SUVmin in SCC are significantly higher than in AC. This is in line with the report of de Geus-Oei et al. [18], who found that FDG uptake was significantly higher in SCC than in AC. Many factors can influence the extent of FDG uptake, such as the expression level of the glucose membrane transporters, GLUT-1 and GLUT-3 and tumor cell differentiation. The underlying mechanisms for FDG accumulation in tumors are complex [18]. For example, Brown et al. found that GLUT-1expression was higher in SCC than in AC [19]. And, Lee et al. reported that EGFR-overexpression status was significantly associated with higher SUVmax on FDG-PET [12].

We observed significantly higher Ktrans and Ve in AC than in SCC (P = 0.035 and 0.042 respectively), but not in Kep and iAUC (P = 0.25 and 0.069 respectively). At first the tumor cells are nourished by diffusion. When they reach a certain size, tumor angiogenesis factors are secreted and tell the recipient bed to start the formation of a vascular plexus supplying the growing tumor. Ktrans does not simply represent vascular leakiness but reflects delivery of contrast to the extravascular space. The increased Ktrans in the observed tumors may imply increasing drug delivery, which, in turn, results in a treatment response. Although there are no results in the literature regarding the predictive role of Ktrans in the treatment response in NSCLC subtypes, its predictive role in colorectal cancer [20] and colorectal liver metastases have been reported [21]. Ve mainly reflects the cellularity of tumor tissue [22], [23]. Histologically, AC characteristically shows a replacement growth pattern in which cylindric tumor cells grow on the walls of preexisting alveoli, with preservation of the underlying architecture of the lung [24], whereas SCC shows a largely solid growth pattern, with malignant cells proliferating compressively and expansively without a replacement growth pattern [25]. The growth pattern difference to some extent causes that the cellularity of SCC is significantly higher than that of AC, and further manifest as the difference in the Ve.

We found that tumor vascularity and glucose metabolism negatively correlated in AC, but not in SCC. The correlations between tumor vascularity and glucose metabolism in NSCLC in the previous reports are conflicting. Hunter et al. observed a positive correlation between glucose metabolic rate and permeability-surface area product, but not with the extracellular contrast distribution space [26]. Similar results were obtained by using dynamic CT [27]. However, no correlation was found in Hoekstra [28] and Sauter’s [29] reports. Miles’s study suggested that blood flow–metabolic relationships are dependent on tumor size in NSCLC [30]. In these studies, some included relative little samples [26], [28], [30]; and the implemented modality to evaluate the tumor vascularity were also different, including dynamic T1-weighted magnetic resonance imaging [26], [30], H_(2)_^(15)^O and positron emission tomography [28], dynamic CT [27], and volume perfusion CT [29]. So a direct comparison of our results with the initial reports on the correlation between SUV and tumor perfusion cannot be performed. Additionally, all these studies considered NSCLC as an entity, and none separate AC and SCC in lung and respectively analyzed. In fact, AC and SCC in lung are heterogeneous in many clinical aspects.

The data from the current study importantly showed that the metabolism-vascularity relationship varies with histologic subtypes of NSCLC, with AC having a negative correlation, but not in SCC. Shastry et al [31] found that low metabolism with high vascularity was a feature of AC whilst high metabolism with high vascularity was a feature of SCC. The findings of the current study reinforce their previous findings. Adenocarcinomas were more likely to have low metabolism and high vascularity, which could partly account for the negative correlation in AC in our results.

Previous studies using PET tracers have also found that the balance between blood flow and metabolism may provide prognostic/predictive information. Although it is reasonable to hypothesize that the metabolic requirements of tumors are mirrored by alterations in tumor haemodynamics, an association between mismatched tumor blood flow and metabolism and adverse tumor biology has been illustrated by many studies [32]–[35]. Aronen et al. [32] found that uncoupling of vascularity and metabolism was a feature of high-grade gliomas; Mankoff et al. [33] showed that breast cancers with a high ratio of glucose metabolism to perfusion were less likely to respond favorably to treatment; Komar at al. [34] observed that both malignant and benign pancreatic lesions were associated with decreased perfusion, and in patients with malignant diseases, a high ratio of metabolism to blood flow seemed to predict poor survival. Mankoff at al. commented on the work of Komar that although tightly coupled in most normal tissues, blood flow and metabolism are often not well matched in tumors. A flow-metabolism mismatch, specifically, high metabolism relative to blood flow, can be recognized in tumors by functional and molecular imaging and is associated with poor response to treatment and early relapse or disease progression. We speculated that the decorrelation of tumor vascularity and glucose metabolism may underlie the relative poor outcome in SCC of lung in Chinese [3]. It would be interesting to investigate whether the metabolism-vascularity relationship may predict the prognosis, and as such, a longitudinal study could be useful.

Some limitations should be mentioned in our study. The presence of hypoxia in the tissue microenvironment which may increase the FDG-SUV, the microscopic necrotic foci with reduced DCE-MRI parameters, the potential influence of tumor heterogeneity in large tumors that may contain some latent metabolic potential, the presence of partial volume effects that may hamper the DCE-MRI and SUV measurements, the limitations of the applied pharmacokinetic model [36] and the static SUV estimation in the metabolic phase are possible limiting factors in the validation of the association between the DCE-MRI parameters and the metabolic activity of lung cancer. To confirm the relationship between the uncoupling of tumor vascularity and glucose metabolism and clinical outcome in NSCLC subtypes, longitudinal study is needed. Given the effects of respiratory movement, although we have taken measures in the DCE-MRI data acquisition and processing procedure, we couldn’t completely overcome the confounding.

In conclusion, AC and SCC showed different patterns in both tumor vascularity and glucose metabolism. Tumor vascularity and glucose metabolism correlated in AC, but not in SCC. These differences may underlie the heterogeneity in clinical aspect of NSCLC subtypes and have implications for their imaging profiling and monitor the treatment response.
